# Comment on ‘Role of Evogliptin for Intra‐Class Therapeutic Interchange in Patients With Type 2 Diabetes Mellitus: Subgroup Analysis From A Multi‐Centre, Prospective, Observational Study (REVISE Study)’

**DOI:** 10.1002/edm2.70289

**Published:** 2026-07-17

**Authors:** Aman Batool Hashmi, Syeda Bareera, Muhammad Faizan Khan

**Affiliations:** ^1^ Khyber Girls Medical College Peshawar Pakistan; ^2^ Khyber Medical University Peshawar Pakistan; ^3^ Nowshera Medical College Nowshera Pakistan

To the Editor,

1

We read with great interest the recent article by Lee et al. [1] The authors should be commended for addressing an important clinical question regarding intra‐class therapeutic interchange (ICTI) in patients with inadequately controlled type 2 diabetes mellitus. Their findings provide valuable real‐world evidence supporting the potential role of evogliptin as a therapeutic option. However, we would like to highlight several methodological considerations that may influence the interpretation of the reported findings.

Our main concern is regression to the mean, which is one of the major sources of bias in ‘before and after’ observational studies. The participants were included only if baseline HbA1c was ≥ 7.0%, thus results were based on a raised clinical measurement. Extreme values will typically become closer to the average on later attempts to measure, even if the attempt is not a real result of the intervention [2]. This is a significant issue since baseline HbA1c was the most significant single predictor of HbA1c reduction in the multivariable model at 24 weeks (*β* = −0.4895, *p* < 0.0001) [1]. The HbA1c reduction seen may be due to natural biological variation, increased follow up, or to measurement variability, in the absence of a concurrent comparator group still on the original DPP‐4 inhibitor [1, 2].

Another important consideration is confounding by indication. Patients were switched to evogliptin due to clinical reasons, but in the absence of sufficient detail and consideration of treatment allocation analysis, the reasons for switching were not described [1]. Of course, in routine clinical practice, physicians may be more inclined to switch patients who differ in how much they adhere to the treatment, willingness to change lifestyle, disease trajectory, presence of co‐morbidities or risk of follow‐up. These factors are not measured and can have independent effects on glycemic outcomes. The authors have controlled for a number of baseline factors such as prior DPP‐4 inhibitor type, age, sex, BMI, diabetes duration, eGFR, baseline HbA1c and concomitant medication use, but there may be residual confounding [1]. In this observational context, treatment‐selection bias could be reduced and causal inference could be strengthened by the use of propensity score methods and/or target‐trial emulation [3, 4].

In addition, findings of subgroups should be interpreted with caution. The authors found that patients receiving a switch from sitagliptin experienced the largest mean HbA1c reduction at 24 weeks, −0.6% ± 0.9% (*p* < 0.0001) and 57.2% of patients had an HbA1c < 7.0% at 24 weeks [1]. But the number of patients in each of these groups was not equal (sitagliptin, 159 patients; alogliptin, 32; anagliptin, 56), which may limit the precision in the smaller groups [1]. Based on these findings, the authors suggest that the differences in the effect between the DPP‐4 inhibitors may be due to pharmacological differences, but a formal interaction effect should be performed prior to making any definitive conclusion about any differences in treatment response [5]. In the multivariable model, only prior anagliptin use was associated with a significantly smaller reduction in HbA1c compared with sitagliptin (*β* = 0.5320, *p* = 0.0015) with the other DPP‐4 inhibitors showing no significant differences [1]. Thus, the subgroup results must be used as hypotheses to test rather than as proof of a clinically relevant difference between the different DPP‐4 inhibitors [5].

In conclusion, the REVISE follow‐up analysis provides useful real‐world evidence suggesting that ICTI to evogliptin may improve glycemic control in patients with inadequately controlled T2DM. The effects observed, however, may have been confounded by indication or been affected by regression to the mean and exploratory subgroup interpretation. Future comparator‐controlled studies with appropriate adjustment for treatment‐selection bias and predetermined interaction testing are required to further elucidate the independent impact of ICTI and to delineate the patients who are most likely to benefit.

## Author Contributions

**Syeda Bareera:** writing – review and editing. **Muhammad Faizan Khan:** supervision. **Aman Batool Hashmi:** conceptualization, investigation, writing – original draft, methodology, validation, visualization, writing – review and editing, software, formal analysis, project administration, data curation, supervision, resources.

## Funding

The authors have nothing to report.

## Ethics Statement

The authors have nothing to report.

## Consent

The authors have nothing to report.

## Conflicts of Interest

The authors declare no conflicts of interest.

## Data Availability

The authors have nothing to report.
